# Diagnostic Value of Systemic Immune Inflammation Index in Identifying Complicated Acute Appendicitis Cases

**DOI:** 10.7759/cureus.73046

**Published:** 2024-11-05

**Authors:** Mehmet Sait Berhuni, Hüseyin Yönder, Hasan Elkan, Muhammed Hamza Koyuncu, Ferhat Özgül, Faik Tatlı, Abdullah Özgönül, Ali Uzunköy

**Affiliations:** 1 General Surgery, Harran University, Şanlıurfa, TUR; 2 General Surgery, Harran, Şanlıurfa, TUR

**Keywords:** acute appendicitis, complicated acute appendicitis, gangrenous appendicitis, perforated appendicitis, phlegmonous appendicitis, systemic immune inflammation index

## Abstract

Purpose: Systemic immune inflammation index (SII) has been used to evaluate the prognosis of various diseases in recent years. This study aimed to investigate the efficacy of SII in the preoperative diagnosis of complicated acute appendicitis (CAA).

Materials and method: The data of patients who underwent surgery for acute appendicitis (AA) between January 2021 and December 2023 at our clinic were retrospectively analyzed. These patients had undergone surgical operations for AA and had available pathology results. Cases with histopathologic findings of gangrenous appendicitis, phlegmonous appendicitis, perforated appendicitis, and periappendicular abscesses were considered CAA. Cases without these manifestations and reported as appendicitis upon histopathological analysis were considered as noncomplicated acute appendicitis(NCAA). Data recorded and evaluated for the study included age, sex, preoperative C-reactive protein (CRP) levels, white blood cell (WBC) count, neutrophil count, lymphocyte count, platelet count, neutrophil-to-lymphocyte ratio (NLR), Alvarado score (AS), SII, and histopathologic examination results.

Results: A total of 441 patients were included in the study. The mean age of the patients was 34.58 ± 11.70 years. There were 200 women (45.35%) and 241 men (54.65%). The number of noncomplicated and complicated cases was 332 (75.28%) and 109 (24.72%), respectively. SII, AS, NLR, and CRP values were significantly higher in the complicated group (p < 0.001, p < 0.001, p < 0.001, p = 0.001, respectively). The sensitivity and specificity of SII in detecting CAAs were 0.624 and 0.607, respectively, with a cutoff value of 1445.

Conclusion: The sensitivity of SII in detecting CAA was 0.624, with specificity and cutoff values of 0.607 and 1445, respectively. SII can be used as an effective parameter for preoperatively predicting whether an AA case is complicated or not.

## Introduction

Acute appendicitis (AA) is characterized by a series of pathophysiological events, including obstruction of the appendix lumen, decreased blood flow to the appendix, impaired mucosal barrier function, bacterial invasion, inflammatory cell infiltration, tissue hypoxia, necrosis, and perforation [1,2]). AA is the most prevalent etiology of acute abdomen requiring surgical treatment. Appendectomy is one of the most frequently performed emergency surgical procedures by general surgeons, with a mortality rate of 7-70 per 1,000 cases [3].

The diagnosis of AA relies on a combination of clinical, physical examination, laboratory, and radiologic findings, which can sometimes be challenging. Notably, physical examination may prove to be misleading, especially if the patient is administered with analgesics. CT is the most frequently used radiological method for the purposes of diagnosis [4]. However, CT has been associated with undesirable outcomes, including increased radiation exposure and economic burden during diagnosis. Therefore, new biomarkers and scoring systems are required for the diagnosis of AA [4,5]. The Alvarado score (AS), defined by Alfredo Alvarado in 1986, provides practical diagnostic support for AA [6]. The neutrophil-to-lymphocyte ratio (NLR) is another diagnostic marker used to assess disease severity, with several studies reporting its effectiveness. Recent research by Eun et al. suggests that, in addition to its diagnostic value, NLR could help decide the preferred imaging technique for AA [7].

Antibiotic therapy-based medical treatment has been proposed as an alternative to surgery, particularly for uncomplicated AA [8,9]. Thus, it is crucial to preoperatively determine whether a case is complicated or not.

The systemic immune inflammation index (SII), first defined by Hu in 2014, is a simple and low-cost index reflecting the balance between inflammation and immune response [10,11]. Hu used SII to evaluate prognosis in patients with hepatocellular carcinoma after curative resection, reporting that high scores were indicative of poor prognosis [10]. Recent studies have suggested that SII is a reliable indicator of inflammation and can be used to diagnose various diseases, including neurological disorders, cancers, and chronic inflammatory diseases, and predict their prognosis [2,12-14].

In the context of AA, complicated AA (CAA) includes periappendicular abscess, phlegmon, gangrenous appendicitis, and appendiceal perforation. Morbidity and mortality are heightened in CAA, particularly due to the risk of peritonitis and sepsis following appendiceal perforation. Accurate preoperative diagnosis of CAA is crucial for guiding surgical treatment [15]. Preoperative recognition of CAA cases based on SII may help surgeons perform appropriate treatment.

This study aimed to investigate the predictive value of SII in the preoperative diagnosis of CAA.

## Materials and methods

Study design, setting, and patient population

The study was a retrospective review of patients who underwent surgery for AA between January 2021 and December 2023 at our clinic. Electronic media records were used for data collection. The study adhered to the principles stipulated in the World Medical Association Declaration of Helsinki-Ethical Principles for Medical Research Involving Human Subjects. Approval was obtained from Harran University Clinical Research Ethics Committee (13.05.2024/06) prior to the commencement of the study. Patients diagnosed with AA who had undergone surgery and had available pathology results were included. Cases with histopathologic findings of gangrenous appendicitis, phlegmonous appendicitis, perforated appendicitis, and periappendicular abscess were classified as CAA. Laboratory values from admission were used to calculate the SII and NLR. The AS was calculated using the patient’s examination form and laboratory data at the time of presentation to the emergency department. In this retrospective study, examinations were performed by different professionals, which was considered a limitation associated with consistency in the calculation of AS. Exclusion criteria included patients under 18 years of age, those with inflammatory bowel disease, pregnant or lactating women, septic patients, those with unstable vital signs or shock, and patients with a history of cancer, known hematologic diseases, normal appendix (noninflamed) or histopathology, appendiceal tumors, and mucoceles.

Data collection and calculation

The following data were recorded and evaluated: age, sex, preoperative C-reactive protein (CRP) level, white blood cell (WBC) count, neutrophil count, lymphocyte count, platelet count, NLR, AS, SII, and histopathologic examination results. The laboratory data used in the calculation of SII in the study were retrieved from the data obtained from the first blood samples taken at admission. Secondary tests or postoperative tests intended for control purposes were not used in the calculations.

SII was obtained by dividing the neutrophil count by the lymphocyte count and multiplying the result by the platelet count. NLR was calculated by dividing the neutrophil count by the lymphocyte count. The AS was derived from anamnesis, physical examination, and laboratory data at the time of presentation to the emergency department.

Statistical analyses

Statistical analyses were conducted using SPSS for Windows, version 25.0 (IBM SPSS Inc., Chicago, USA). The Shapiro-Wilk test was employed to assess the normality of data distribution. Given that the numerical variables did not follow a normal distribution, the data were summarized as median values with interquartile ranges (IQRs). The comparison of numerical variables between two groups was performed using the Mann-Whitney U test, appropriate for non-normally distributed data. Receiver operating characteristic (ROC) analysis was employed to determine the optimal cut-off values for the SII, NLR, and AS ratio within the CAA group. All analyses were conducted with a 95% confidence interval, and statistical significance was defined as a two-tailed p-value of less than 0.05.

## Results

A total of 441 patients were included in the study. The mean age of the patients was 34.58 ± 11.70 years. There were 200 women (45.35%) and 241 men (54.65%). Of these, 332 patients (75.28%) had noncomplicated AA (NCAA), and 109 patients (24.72%) had CAA.

There was no significant difference in age between the two groups (p = 0.167). Although WBC values were higher in CAA cases, this difference was not statistically significant (p = 0.102). CRP values were significantly higher in CAA cases (p = 0.001). SII, AS, and NLR values were all significantly higher in the CAA group compared to the NCAA group (p < 0.001 for each). The median SII values for the CAA and NCAA groups were 1783.70 (1734.70) and 1205.40 (1396.00), respectively. Patients in the CAA group had significantly longer hospital stays compared to the NCAA group (p < 0.001) (Table 1).

**Table 1:** A comparison of CAA and NCAA groups CAA: Complicated acute appendicitis; NCAA: Noncomplicated acute appendicitis; SII: Systemic immune inflammation index; NLR: Neutrophil-to-lymphocyte ratio; AS: Alvarado score; CRP: C-reactive protein; WBC: White blood cell; IQR: Interquartile range ٭p values indicate significance

Variables	CAA	NCAA	p value
Age (years), median (IQR)	31.00 (23.50)	30.50 (16.00)	0.167
Hospital stay (days), median (IQR)	2.00 (2.00)	2.00 (1.00)	˂ 0.001٭
SII, median (IQR)	1783.70 (1734.70)	1205.40 (1396.00)	˂ 0.001٭
NLR, median (IQR)	7.60 (6.15)	4.90 (5.60)	˂ 0.001٭
AS, median (IQR)	8.00 (1.00)	6.00 (1.00)	˂ 0.001٭
CRP, median (IQR)	5.07 (13.61)	2.53 (6.86)	˂ 0.001٭
WBC, median (IQR)	13.60 (7.47)	12.63 (5.65)	0.102

**Figure 1:**
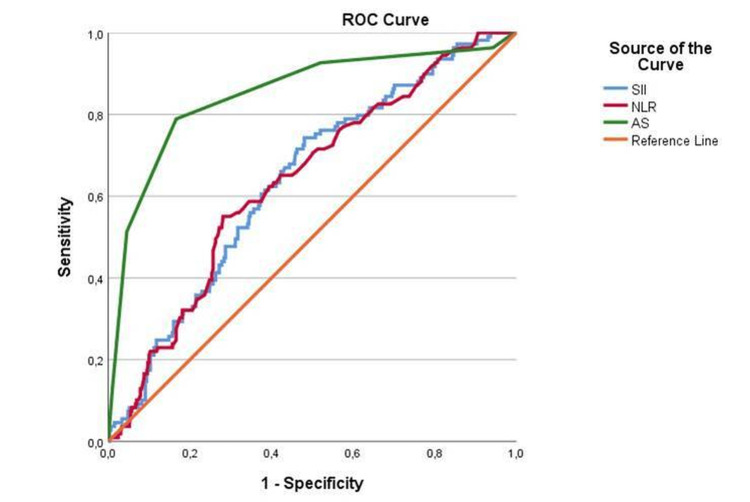
ROC curve of biomarkers to predict complicated appendicitis ROC: Receiver operating characteristic

ROC analysis was performed to determine the effectiveness of SII in diagnosing CAA. The sensitivity and specificity of SII for detecting CAA were 0.624 and 0.607, respectively, with a cutoff value of 1445. This means that SII values above 1445 can be recognized as significant CAA cases. For NLR, the sensitivity and specificity were 0.615 and 0.610, respectively, with a cutoff value of 6.05. The AS had a sensitivity of 0.789 and specificity of 0.844 for distinguishing CAA, with a cutoff value of 6.50 (Table 2 and Figure 1).

**Table 2:** ROC analysis results ROC: Receiver operating characteristic; SII: Systemic immune inflammation index; NLR: Neutrophil-to-lymphocyte ratio; AS: Alvarado score; AUC: Area under the curve; CI: Confidence interval; ¥: Mann–Whitney U test ٭ p values indicate significance

Parameter	Cut-off value	Sensitivity	Specificity	AUC	Lower bound (%95 CI)	Upper bound (%95 CI)	p value^¥^
SII	1445	0.624	0.607	0.638	0.581	0.696	<0.001^٭^
NLR	6.05	0.615	0.610	0.637	0.579	0.695	<0.001^٭^
AS	6.50	0.789	0.844	0.850	0.803	0.897	<0.001^٭^

## Discussion

AA affects all age groups but is more prevalent in men and is commonly associated with acute abdomen [16]. Despite the use of various diagnostic tools, including ultrasonography (USG), CT, scoring systems, and physical examinations, the misdiagnosis rate for AA remains high, up to 15% [17]. A significant challenge is the inability to determine preoperatively whether AA is complicated or not, which complicates treatment decisions and patient counseling. Certain previous studies suggested that it was important to know preoperatively whether an AA case was complicated or not, both in terms of surgeon’s decision on the treatment modality and informing the patient about possible outcomes [15,18]. While NCAA cases can often be treated with appendectomy, CAA cases, especially those associated with abdominal sepsis, pose life-threatening risks [19]. Traditional diagnostic approaches combine history, physical examination, laboratory tests, and radiologic imaging. However, radiologic methods have their limitations, such as the operator-dependence of ultrasound, the radiation risk of CT, prolonged diagnostic processes, and high costs. Thus, there is a need for more accurate, cost-effective, rapid, and reliable diagnostic tools AA [11].

A study by Şener et al. compared 150 AA cases with a group of 150 control patients, who presented to the emergency department with abdominal pain. The sensitivity and specificity of SII for the diagnosis of AA were reported as 82% and 66.7%, respectively. The same study compared 18 complicated cases of 150 AA patients with 132 uncomplicated cases, and it was reported that SII could detect CAA cases with 88.9% sensitivity and 88.9% specificity [11]. Tekeli et al. reported that SII values were significantly higher in CAA cases among pediatric patients [20]. Additionally, Cakcak et al. compared AA cases prior to the pandemic with AA cases during the pandemic in adult patients and reported that complicated cases were more prevalent during the pandemic and that SII were significantly higher in patients with CAA [21]. One possible reason for the higher number of CAA cases in this study could be the delayed diagnosis process due to patients' hesitation to present to the hospital during the pandemic. It is likely that the prolonged diagnostic process leads to increased tissue destruction and more severe stimulation of inflammatory mechanisms. Consistently, SII were significantly higher in patients with CAA in the present study. The sensitivity and specificity of SII in detecting CAA were 0.624 and 0.607, respectively, with a cutoff value of 1445. The sensitivity and specificity of SII appear to be lower than CT. However, in cases where CT scan isn’t possible and in cases with suspicious findings on CT, SII can be used as a supportive diagnostic marker.

Dinç et al. reported that WBC values were statistically significantly higher in CAA cases compared to NCAA cases [18]. Zhang et al. reported that WBC, CRP, and NLR values were significantly higher in CAA cases compared to NCAA cases in a study of 1,514 patients at two centers [22]. Tekeli et al. also investigated pediatric patients and reported that CAA cases had significantly higher CRP, WBC, and NLR values compared to NCAA cases [20]. Hajibandeh et al. suggested that NLR was a significant predictor of CAA cases upon in a meta-analysis [23]. Although Haak et al. suggested that AS was limited in preoperative detection of CAA and NCAA in their study, Cakcak et al. reported that AS values were significantly higher in CAA cases compared to NCAA cases in their study [21,24]. In the present study, CAA patients had significantly higher AS, NLR, and CRP values compared to patients in the NCAA group. Although WBC levels were higher in the CAA group, this difference was close to but not statistically significant. Indices such as SII and NLR can be used to support imaging methods with current data. However, if the reliability of these indices is demonstrated in future studies, they can be used as an alternative to imaging methods.

There were some limitations in our study. The first of these can be seen as low number of cases. Our findings can be interpreted more accurately with studies involving larger case series. Another limitation was that the study is single-centered. Since the results can’t be generalized with the findings of one center, our findings need to be supported by multicenter studies. Finally, the retrospective nature of our study can be seen as another limitation. 

## Conclusions

In conclusion, SII is an effective, reliable, and valuable parameter for the preoperative diagnosis of CAA. Accurate differentiation between NCAA and CAA using SII can aid surgeons in providing better-informed treatment plans and managing patient expectations. We believe that since SII doesn’t cause any additional costs, it may be useful to use it as a diagnostic marker together with current diagnostic methods. Future research should include prospective multicenter studies with larger sample sizes to support the clinical adoption of SII.
